# Peer coaching in cardiac surgery: a pilot study on rehabilitation participation and perioperative challenges

**DOI:** 10.1093/icvts/ivae219

**Published:** 2024-12-26

**Authors:** Kanwar Parhar, Aaron Holm, Ravi S Hira, Lara Oyetunji, Jeannie Collins-Brandon, Eric J Lehr, Sarah Speck

**Affiliations:** Department of Cardiology, Elson S. Floyd College of Medicine, Spokane, WA, USA; Aaron Holm, Executive Director, Patient Circle Research Institute, Issaquah, WA, USA; Pulse Heart Institute and MultiCare Health System, Tacoma, WA, USA; Cardiac Care Outcomes Assessment Program (COAP) and Foundation for Health Care Quality, Seattle, WA, USA; Department of Surgery, University of Washington School of Medicine, Seattle, WA, USA; Cardiac Care Outcomes Assessment Program (COAP) and Foundation for Health Care Quality, Seattle, WA, USA; Division of Cardiac Surgery, Swedish Heart and Vascular Institute, Seattle, WA, USA; Division of Cardiac Surgery, Swedish Heart and Vascular Institute, Seattle, WA, USA

**Keywords:** Cardiac rehabilitation, Peer coaching, Perioperative challenges, participation rates

## Abstract

Following cardiac surgery, active participation in cardiac rehabilitation (CR) is associated with reduced cardiovascular events and improved survival. However, CR attendance remains persistently low, with only ∼25% of patients participating. The Peer Coaching for Cardiac Patients (PCCP) pilot programme aimed to assess whether peer coaching could enhance CR participation and reduce perioperative anxiety and depression in cardiac surgery patients. Ten patients scheduled for elective cardiac surgery were enrolled, receiving 4 60-min coaching sessions via Zoom, by a coach who had undergone coronary artery bypass grafting in the past. Outcomes were measured by CR participation rates, Patient Health Questionnaire (PHQ)-9 scores, and a post-programme survey. Seven of the 10 patients completed the PCCP programme, all of which attended CR for an average of 19.3 ± 8.70 sessions and 9.57 ± 3.0 weeks. No statistically significant difference in PHQ-9 scores was observed (*P* = 0.341). Participants rated the programme highly in its role in anxiety reduction (9.0 ± 1.2) and likelihood of participating in CR (9.43 ± 1.05). These results suggests that peer coaching shows potential to support CR participation and address perioperative anxiety and depression. Future studies with larger sample sizes, well-defined control groups and extended follow-up are warranted to validate these preliminary findings.

## INTRODUCTION

Cardiovascular disease is widely prevalent and remains the leading cause of death globally, with ∼1.5 million undergoing cardiac surgery annually [1]. Many patients face considerable psycho-social stressors and reduction of quality of life after cardiac surgery [2]. Perioperative anxiety and depression are 2 prevalent issues that are linked with poor recovery and have been strongly associated with negative health outcomes [3].

To address these issues, cardiac rehabilitation (CR) programmes were developed as comprehensive interventions with structured physical activity, patient education and psychological support aimed at promoting recovery following cardiac surgery [4]. Active participation in CR has been linked to a reduction in subsequent cardiovascular events and better survival with a class 1 recommendation from the American Heart Association guidelines for management of patients undergoing cardiac surgery [4]. However, despite the known benefits participation remains persistently low with only ∼25% of patients participating [5]. The barriers to participation in CR are multifaceted, with the major ones being a lack of physician endorsement, patient time constraints, transportation issues and cost [6].

Given the benefits of CR with low participation rates, new interventions are needed to increase patient participation. The effect of peer coaching by individuals who have themselves undergone cardiac surgery on CR utilization and psychological well-being of patients has not been previously studied. This study aims to evaluate the feasibility and potential benefits of a peer coaching programme for patients undergoing elective cardiac surgery. We hypothesized that the programme would enhance CR participation and reduce perioperative anxiety and depression.

## METHODS

### Study patients

Ten patients scheduled for elective cardiac surgery at one site were offered the Peer Coaching for Cardiac Patients (PCCP) programme. To mitigate the risk of bias, consecutive patients received an informational flier with the contact information of the lead programme coach. The first 10 patients who met the programme’s inclusion and exclusion criteria were included in the study. Inclusion criteria: age >21, ability to participate in Zoom coaching and elective surgery at least 14 days after enrollment for presurgery coaching. Exclusion criteria: emergency surgery, inability to communicate effectively in English and no access to Zoom. After reviewing the programme’s information sheet, patients first verbally consented to be contacted by the coach for a 30-min Zoom session to discuss the programme in detail. Verbal and written informed consent were then obtained prior to beginning and for use and collection of data in an anonymous manner. IRB approval was not required, as this pilot study does not contribute to generalizable knowledge.

### PCCP programme details

The programme was led by coach A.H. who completed the International Coaching Federation accredited training and had undergone prior coronary bypass surgery. The programme ran from November 2023 to April 2024. The sessions were free and no payments were made to the coach. Each participant attended 4 60-min coaching sessions via Zoom, 2 before surgery and 2 after (see Supplementary Material, S1 for session breakdowns) [7]. The decision to conduct peer coaching via Zoom was guided by several factors: it enabled continuous support across the surgical journey within patients’ homes, eliminated travel costs and minimized infection risk during recovery.

### Surveys and outcomes

Data collection included patient demographics and CR enrollment and participation. Pre- and post-programme Patient Health Questionnaire (PHQ)-9 scores and a post-programme survey were also collected. The post-programme survey asked patients to agree or disagree with statements using a rating of 0 (strongly disagree) to 10 (strongly agree). Programme evaluation was done through post-programme written and virtual feedback. Data were collected in a pseudo anonymized manner and stored securely. Patients with missing data were removed from the analysis.

### Statistical analysis

For patients who completed the programme, descriptive statistics were conducted on CR participation duration, number of sessions attended and post-programme survey scores. Continuous variables were represented as mean ± standard deviation. PHQ-9 scores were compared via a paired *t*-test. Statistical analysis was performed using SPSS, version 27 (IBM, Armonk, NY, USA). *P* < 0.05 was considered significant for all comparisons.

## RESULTS

The programme included 10 participants, 5 males, 4 females and 1 who identified as non-binary, aged 48 to 76 years. Of these, 7 patients fully completed the PCCP programme, 1 completed the programme but did not complete the post-programme survey, 1 attended 2 presurgery sessions and 1 patient declined participation after enrollment in the programme.

All the programme participants went on to attend CR for an average of 19.3 ± 8.70 sessions and a duration of 9.57 ± 3.0 weeks. When comparing the pre-programme and post-programme PHQ-9 scores no statistically significant difference was seen (*P* = 0.341). The post-programme survey revealed a mean score of 9.0 ± 1.2 in reducing patients fear and anxiety, all the patients unanimously agreed on recommending the peer coaching programme and mean score of likelihood of the programme resulting in CR enrollment was 9.43 ± 1.05 (Table 1). When asked about the most important aspects of the coaching programme in this survey, unanimous agreement was seen for sharing the lived experience with the coach and having a support network outside their friends/family (Table 2).

**Table 1:** Post-programme survey results for the PCCP programme

	Patient #								
Survey questions	1	2	3	4	5	6	7	Mean ± SD	95% CI
Helpfulness before surgery	10	9	9	10	10	8	10	9.43 ± 0.73	(8.89–9.97)
Helpfulness after surgery	10	9	6	10	10	8	10	9.0 ± 1.41	(7.96–10.04)
Reducing rear/anxiety	10	8	7	10	10	8	10	9.0 ± 1.2	(8.11–9.89)
Number of sessions sufficient?	5	9	7	10	10	8	10	8.43 ± 1.76	(7.13–9.73)
Length of sessions sufficient?	5	9	9	10	10	7	10	8.57 ± 1.76	(7.27–9.87)
Session format sufficient?	10	9	8	8	10	10	10	9.29 ± 0.88	(8.64–9.94)
Would recommend coaching?	10	10	10	10	10	10	10	10.0 ± 0.0	(10.00–10.00)
Likelihood of coaching making you join a CR programme	10	9	10	7	10	10	10	9.43 ± 1.05	(8.65–10.21)

**Table 2:** Patients ratings on the most important aspects of PCCP coaching

Experience	Percentage of patients that agreed
Sharing lived experience	100% (7/7)
Learning management techniques	71% (5/7)
Addressing my emotions	86% (6/7)
Setting surgery and recovery expectations	86% (6/7)
Having a support network outside family	100% (7/7)

## DISCUSSION

Our findings suggest that the PCCP programme is feasible and could improve CR participation in cardiac surgery patients. In this small pilot study, there was no significant difference in depression scores before and after programme participation. Post-programme survey scores suggest participants found the programme to be beneficial in reducing fear and anxiety and would recommend the programme to other patients.

Low participation in CR is a well-documented issue despite its strong association with improved outcomes [3, 4]. Studies have consistently shown that only ∼25% of patients nationally attend CR following surgery, raising concerns about gaps in post-operative recovery and care [8]. In our study, 7 out of 10 enrolled patients completed the PCCP programme and all went on to participate in CR. Long-term benefits of cardiac surgery are strongly correlated with active CR participation [4]. Furthermore, patients in our study reported benefits of peer coaching in managing perioperative anxiety, which could support future studies exploring peer coaching as a resource for recovery.

The success of peer coaching in improving participation has been well documented in other fields of medicine as well, Simon *et al.* [9], demonstrated peer coaching to increase participation and retention in a bipolar disorder recovery programme. In our study, participants rated the programme characteristics highly, with unanimous agreement in recommending the coaching programme (10.0 ± 0). The most valued aspect by patients were the coach sharing lived experience with them and having support network outside their family and friends (Table 2). One patient shared, ‘It helped a lot to be able to talk to somebody who had a similar procedure done and know what to expect’. This highlights that peer interactions may help normalize patient experiences and provide emotional support during recovery. Additionally, the virtual format of the sessions was well-received, with a mean score of 9.29 ± 0.88. Patients went on to say, ‘The best thing is you don’t have to travel, it’s easier on the schedule and easier on the lifestyle’. In line with this, recent work by Scheenstra *et al.* [10] demonstrated the effectiveness of telerehabilitation in reducing adverse events following elective cardiac surgery, further supporting the potential of virtual interventions to enhance patient outcomes.

This study has several limitations. The small number of participants, lack of diversity and single coach limits the generalizability of these findings. Additionally, without a control group, our study cannot establish comparative effectiveness of peer coaching relative to standard or alternative interventions. Additionally, employing other objective methods to assess patient feedback and anxiety may have yielded different results. Finally, extending the follow-up period will allow for an evaluation of the long-term effects of the programme on psychological well-being, CR participation and cardiovascular health.

## CONCLUSION

Our pilot study suggests that peer coaching in patients undergoing cardiac surgery is feasible and shows potential to address perioperative psychological challenges and improve CR participation rates. Future studies with larger, more diverse and longer follow-up patient populations are warranted to confirm these preliminary findings and explore the broader applicability of peer coaching in cardiac surgery patients.

## Supplementary Material

ivae219_Supplementary_Data

## Data Availability

Data available upon request.
