# Supplementary material for: Peer coaching in cardiac surgery: a pilot study on rehabilitation participation and perioperative challenges
Source: Interdiscip Cardiovasc Thorac Surg. 2024 Dec 26;40(1):ivae219. doi: 10.1093/icvts/ivae219 (PMC11717349; doi:10.1093/icvts/ivae219)
Supplement: ivae219_Supplementary_Data [file ivae219_supplementary_data.docx]

Supplement 1: PCCP program coaching session breakdown.

Session #1 – pre-surgery

1. Program overview and introductions.
2. Introduction of guided somatic breathwork for anxiety management and nervous system regulation.
3. Discussion about the patient’s emotional and physical readiness for surgery, as well as their support system.
4. Discussion on daily practices leading up to surgery, with a focus on observing and experiencing anxiety.
5. Writing prompts provided to explore feelings between coaching sessions.

Session #2 – pre-surgery

1. Discussion on the patient’s current feelings and any new updates.
2. Guided somatic breathwork
3. Discussion addressing the patient’s anxiety management techniques for surgery preparation and expectations for post-surgery.
4. Introduction to guided breathwork aimed at creating a positive association between chest sensations and healing.
5. Patients were encouraged to ask questions and consider their support system and home readiness for recovery.
6. Positive association guided breathwork.

Session #3 – post-surgery

1. Discussion about the patient’s current feelings post-surgery.
2. Modified guided somatic breathwork, allowing the patient to breathe at a comfortable depth with a focus on experiencing sensations in the sternal area.
3. Discussion of the patient’s experiences with surgery, including waking up, tube removal, recovery in the hospital, and returning home.
4. Patients were encouraged to express how they would like to be supported and whether they feel comfortable asking for that support.
5. Positive association guided breathwork.

Session #4 – post-surgery

1. Discussion on the patient’s current state several weeks after surgery, reflections on the sessions, and any ongoing concerns.
2. Modified guided somatic breathwork.
3. Discussion of the patient’s recovery experience, their perspective on the surgery, feelings about discussing the surgical experience, and thoughts on moving forward.
4. Discussion regarding the patient’s return to physical activity and plans for cardiac rehabilitation.
5. Positive association guided breathwork.
